# Specific Recognition of p53 Tetramers by Peptides Derived from p53 Interacting Proteins

**DOI:** 10.1371/journal.pone.0038060

**Published:** 2012-05-31

**Authors:** Ronen Gabizon, Tobias Brandt, Shahar Sukenik, Noa Lahav, Mario Lebendiker, Deborah E. Shalev, Dmitry Veprintsev, Assaf Friedler

**Affiliations:** 1 Institute of Chemistry, The Hebrew University of Jerusalem, Jerusalem, Israel; 2 MRC Laboratory of Molecular Biology, Cambridge, United Kingdom; 3 The Wolfson Centre for Applied Structural Biology, The Hebrew University of Jerusalem, Jerusalem, Israel; 4 Laboratory of Biomolecular Research, Paul Scherrer Institut, Villigen, Switzerland; 5 Department of Biology, ETH Zurich, Zurich, Switzerland; Cardiff University, United Kingdom

## Abstract

Oligomerization plays a major role in regulating the activity of many proteins, and in modulating their interactions. p53 is a homotetrameric transcription factor that has a pivotal role in tumor suppression. Its tetramerization domain is contained within its C-terminal domain, which is a site for numerous protein-protein interactions. Those can either depend on or regulate p53 oligomerization. Here we screened an array of peptides derived from proteins known to bind the tetrameric p53 C-terminal domain (p53CTD) and identified ten binding peptides. We quantitatively characterized their binding to p53CTD using fluorescence anisotropy. The peptides bound tetrameric p53CTD with micromolar affinities. Despite the high charge of the binding peptides, electrostatics contributed only mildly to the interactions. NMR studies indicated that the peptides bound p53CTD at defined sites. The most significant chemical shift deviations were observed for the peptides WS100B(81–92), which bound directly to the p53 tetramerization domain, and PKCα(281–295), which stabilized p53CTD in circular dichroism thermal denaturation studies. Using analytical ultracentrifugation, we found that several of the peptides bound preferentially to p53 tetramers. Our results indicate that the protein-protein interactions of p53 are dependent on the oligomerization state of p53. We conclude that peptides may be used to regulate the oligomerization of p53.

## Introduction

Many disease-related proteins exist in equilibrium between active and inactive oligomeric states. The oligomerization equilibrium of these proteins is frequently regulated by post translational modifications [1], binding of ligands [2] and solution conditions such as pH and temperature [3], and plays a vital role in the activity of the protein. Hence, modulating the dynamic nature of oligomerization equilibria to affect protein activity is a promising therapeutic strategy. We previously defined “shiftides” as peptides that bind preferentially to a certain oligomeric state of a protein and shift the oligomerization equilibrium towards it. We have demonstrated this principle for protein inhibition and developed peptides that bound preferentially to the tetrameric state of the HIV integrase protein (IN) and shifted the oligomerization equilibrium towards it. These peptides inhibited the IN enzymatic activity and inhibited HIV replication in cells [4], [5], [6], [7], [8].

In this study we employed the shiftide principle to identify peptides which modulate the oligomerization equilibrium of the tumor suppressor p53. p53 is at the heart of a complex protein network that provides one of the major anti-cancer mechanisms in the cell [9], [10]. It is a transcription factor that is activated and accumulated in the nucleus in response to oncogenic stress. Following its induction, p53 binds specific promoters in the genome and activates the transcription of a wide array of target genes, aimed at eliminating the threat of malignant transformation [9], [10], [11]. p53 is mutated in over 50% of all cancer cases, with the majority of mutations occurring in its DNA-binding core domain [12].

**Figure 1:**
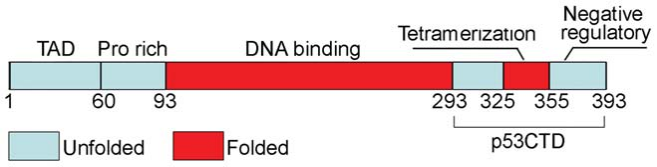
Domain structure of p53. The C-terminal domain contains the folded tetramerization domain (red) flanked by two unfolded domains (cyan). TAD – transactivation domain; CTD–C-terminal domain.

p53 is active as a homotetramer [13] and its tetramerization is mediated by a structurally independent tetramerization domain (p53Tet, residues 326–355) [14], [15] (Figure 1). Tetramerization of p53 is vital to its function and plays a central role in the regulation of p53 activity. The binding of p53 to DNA is highly cooperative both at the level of dimeric p53 [16] and tetrameric p53 [17], and oligomerization-deficient mutants of p53 bind DNA with much lower affinities than the wild type [17]. Moreover, individual p53 core domains and oligomerization-deficient mutants of p53 can bind half-site recognition elements, but these recognition elements are usually not as active as full-site elements in terms of binding and transcriptional activation [18]. Crystallographic studies have shown that isolated p53 core domains assemble into tetramers upon binding full-site recognition elements, with extensive monomer-monomer interactions stabilizing the complex [19]. In addition, the Nuclear Export Signal (NES) of p53 is located within the tetramerization domain and is shielded in p53 tetramers, preventing nuclear export of tetramers of p53 [20]. Full-length p53 has a dissociation constant of ∼20 nM for the dimer-tetramer equilibrium and ∼1.0 nM for the monomer-dimer equilibrium [21].

The p53 C-terminal domain (p53CTD, residues 293–393) is a hub for protein-protein interactions. Many p53CTD-interacting proteins bind specifically to certain oligomeric forms of p53 and thus modulate the p53 oligomerization equilibrium. Within the S100 family, S100B, S100A2, and S100A6 specifically bind tetrameric p53, while S100A1 binds only a monomeric mutant of p53 [22], [23], [24], [25]. Numerous kinases bind monomeric peptides containing parts of the sequence of p53Tet, which are only exposed in p53 dimers or monomers [26], [27], [28]. Binding of proteins from the 14-3-3 family to the p53 negative regulatory domain (p53-NRD, residues 361–393) was shown to activate the DNA-binding of p53 by increasing the tetrameric fraction of p53. The effect was shown to be dependent on the p53 phosphorylation state [29]. Other proteins reported to bind to p53CTD are E3 ligases [30], [31], tumor suppressors [32] and viral proteins [33], [34].

The p53 tetramer has been shown to be stabilized by small molecules. Short poly-arginine peptides bound p53Tet and slightly stabilized the tetramer [35]. The structure of oligomerization deficient mutants of p53Tet could be recovered using rationally designed ligands [36]. Modulation of the oligomerization equilibrium of p53 is also possible by binding to its N-terminal transactivation domain as in the case of p300, which binds the four transactivation domains of p53 simultaneously and stabilizes the tetramer [37].

In this study, ten peptides that bind p53CTD were identified by screening a peptide array containing partially overlapping peptides derived from proteins that bind p53CTD (Table S1) for binding tetrameric recombinant p53CTD. We quantified and characterized the binding of the peptides to p53CTD using fluorescence anisotropy and NMR, tested their effect on the thermodynamic stability of the p53CTD using circular dichroism thermal denaturation measurements, and studied their effect on p53 oligomerization using analytical ultracentrifugation. NMR studies showed that the peptides bound p53CTD at defined sites. Several of these peptides bound specifically to p53 tetramers, and one peptide, PKCα(281–295), caused a mild increase in the thermodynamic stability of the p53CTD tetramer. Our results indicate that the protein-protein interactions of p53 are dependent on the oligomerization state of p53. We conclude that peptides may be used to regulate the oligomerization of p53.

## Results

### Screening of the Peptide Array for Binding p53CTD

**Figure 2:**
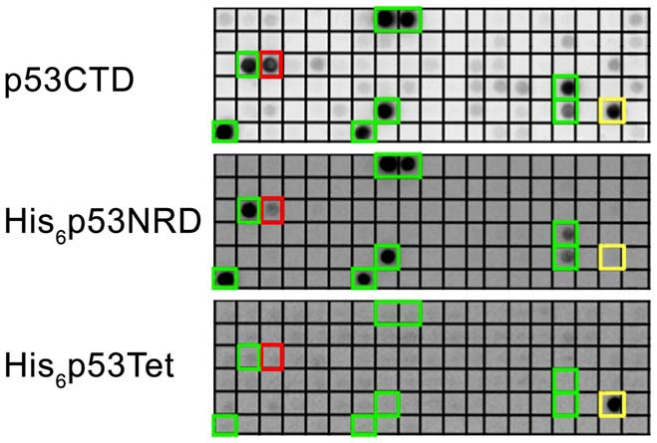
Screening of the peptide array for binding to p53CTD and the constructs p53Tet and p53NRD. Peptides that interacted with p53NRD (p53 361–393) are marked in green. The peptide WS100B(81–92) that interacted with p53Tet is marked in yellow. The peptide PKCα(281–295) that bound more tightly to full-length p53CTD than to p53NRD is marked in red.

We designed an array containing partially overlapping peptides derived from proteins known to bind the p53CTD (for a list of the proteins, see Table S1). We screened the array for binding recombinant p53CTD, which was fully tetrameric at the concentrations used. Screening of the array revealed ten peptides that interacted with p53CTD (Figure 2, Table 1). The p53CTD-interacting peptides were derived from the proteins S100B and S100A4, Protein Kinase C α isoform (PKCα), the E3 ubiquitin ligases Cullin 7 (Cul7) and PARC (Parkin Like Cytoplasmic Protein), and the hsp70 family member Mortalin-2 (Mot2). To identify the precise binding site of these peptides within the p53CTD, we screened the array for binding chemically synthesized His-tagged p53Tet (residues 326–355) and His-tagged p53NRD (residues 361–393), using concentrations of 20 µM, at which p53Tet is tetrameric. Of the ten peptides, nine bound to p53NRD, while the peptide WS100B(81–92) bound to p53Tet (Figure 2, Table 1). The peptide PKCα(281–295) bound to full length p53CTD much more tightly than to p53NRD.

**Table 1:** Binding peptides identified in the peptide array screening.

Name	Binds to:		
	p53(293–393)	p53(361–393)	p53(326–355)
Cul7(376–390)	+	+	−
Cul7(386–400)	+	+	−
PKCα(271–285)	+	+	−
PKCα(281–295)	+	+ (weak)	−
PKCα(641–655)	+	+	−
Mot2(266–280)	+	+	−
S100B(61–75)	+	+	−
S100B(81–92)	+	−	+
S100A4(61–75)	+	+	−
PARC(386–400)	+	+	−

The screening revealed previously unknown binding sites for p53CTD in several proteins. We discovered a single binding site for p53CTD in Mot2, which is located between residues 266–280. It was previously reported that Mot2 binds a region within p53Tet [38], and our peptide array screening revealed that Mot2 also binds at the negative regulatory domain of p53. In PKCα, we identified two binding sites for p53CTD–residues 271–295 and residues 641–655.

### The Peptides Bind Tetrameric p53CTD with Micromolar Affinities

**Figure 3:**
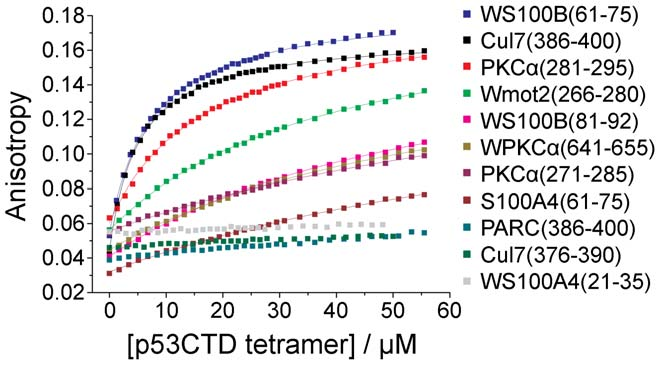
Binding of peptides from the array to p53CTD. p53CTD was titrated into a solution of fluorescein-labeled peptide and the changes in anisotropy were measured. All data were fit to a 1∶1 binding model, indicated by solid lines.

We synthesized the binding peptides, labeled them with fluorescein at their N-termini and used fluorescence anisotropy to validate the array screening results and quantify the binding of the peptides to p53CTD. At the concentrations used, p53CTD was tetrameric [21], [24]. The peptides bound p53CTD with various affinities. The tightest binding peptides Cul7(386–400), WS100B(61–75) and PKCα(281–295) bound p53CTD with dissociation constants of 5–20 µM. The very weak binders Cul7(376–390) and PARC(386–400) showed detectable but non-quantifiable binding (Figure 3, Table 2). The rest of the peptides bound p53CTD with dissociation constants of 20–100 µM. WS100A(21–35), which did not interact with p53CTD in the array, also showed no change in anisotropy. The binding of all peptides fit well to a 1:1 binding model, indicating that the peptides bound a single entity in solution, the existing tetrameric state of p53CTD, with no change in the oligomerization state upon binding.

**Table 2:** Binding affinities of peptides from the array to p53CTD and their effect in AUC*^A^*.

Peptide	Sequence	*K*_d_ (µM*^B^*)	Charge*^C^*	AUC effect
Cul7(386–400)	LDDYEEISAGDEGEF	5.3±0.1*^D^*	−7	Tetramer shift + high order oligomer
WS100B(61–75)	WLDNDGDGECDFQEFM	7.5±0.2	−6	Tetramer shift + high order oligomer
PKCα(281–295)	EEGEYYNVPIPEGDE	17±1	−6	Tetramer shift + high order oligomer
Wmot2(266–280)	WSTNGDTFLGGEDFDQ	41±1	−4	Tetramer shift + high order oligomer
WS100B(81–92)	WVTTACHEFFEHE	61±2	−3	Tetramer shift + high order oligomer
WPKCα(641–655)	WDQLVIANIDQSDFEG	65±3	−4	N/D: No sedimentation observed
PKCα(271–285)	ASGWYKLLNQEEGEY	105±9	−2	No effect
S100A4(61–75)	NLDSNRDNEVDFQEY	98±7	−4	Tetramer shift + high order oligomer
PARC(386–400)	GMRVRMLDDYEEISA	>400*^E^*	−2	Tetramer shift
Cul7(376–390)	TLQPGMRVRMLDDYE	>800*^E^*	−1	Tetramer shift

*A:* All peptides were amidated at their C-terminus.

*B:* The dissociation constant is given in µM tetramer.

C: The charge is estimated for pH = 7.0.

*D:* The error indicated is the standard error obtained from the fit for a representative titration.

*E:* For Cul7(376–390) and PARC(386–400), a rough estimate of the dissociation constants was obtained by fixing the anisotropy amplitude at 0.13, since the amplitude was 0.125–0.140 for all peptides.

### The Binding of the Peptides to p53CTD is Specific and Only Partly Electrostatic

We selected the three tightest binding peptides, Cul7(386–400), WS100B(61–75) and PKCα(281–295), and analyzed the effect of ionic strength on their binding to p53CTD according to equation 1 [39]:
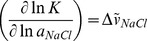
(1)with *K* being the equilibrium constant, *a_NaCl_* the activity of NaCl, and 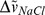 the amount of Na^+^ and Cl^−^ ion pairs released to the solution upon binding of the peptide to the protein. The number of released ions is indicative of the contribution of electrostatic forces to the interaction.

**Figure 4:**
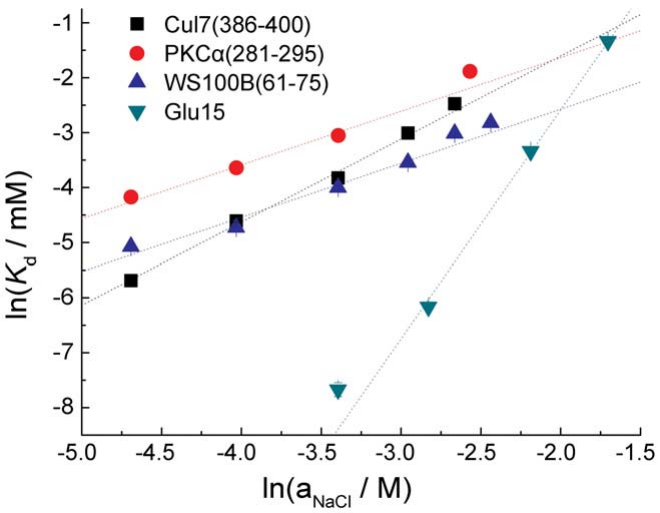
Ionic strength-dependent binding of the peptides to p53CTD. ln(*K*_d_) is plotted vs. ln(NaCl activity) for the tightest binding peptides. The data were fit to a linear model.

Fluorescence anisotropy experiments were conducted on each of the three tightest binders in the presence of increasing NaCl concentrations, and analyzed according to equation 1. The binding affinity decreased with increasing ionic strength, as shown in figure S1 for Cul7(386–400). The obtained dissociation constants were plotted against NaCl solution activity [40] (Figure 4). All the peptides tested exhibited a linear dependence, indicating a contribution of ion release to the binding energy. The slope of the curves was moderate and indicated the release of roughly one ion-pair upon binding. A control peptide, Glu15, consisting of a poly-glutamate chain with 15 residues released about four times more ion pairs than the other peptides tested (Table 3). This number correlates well with the total positive charge of p53CTD (+5). This may indicate that while the negative charge is important in directing the ligand towards the binding site, the actual binding of the three strongest binding peptides is only mildly assisted by electrostatics, and the main contributions to binding come from more specific, close-range interactions. Extrapolation of the data to physiological ionic strengths gave dissociation constants of 15–50 µM for all three peptides (Table 3).

**Table 3:** Number of dissociated ion pairs upon peptide binding to p53CTD.

Peptide	Charge at pH = 7	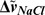	Estimated physiological *K*_d_/µM
Cul7(386–400)	−7	1.51±0.07*^A^*	48±14
PKCα(281–295)	−6	0.98±0.12	48±29
WS100B(61–75)	−6	0.98±0.09	19±7
Glu15	−15	4.17±0.25	17±13

*A:* The error indicated is the standard error obtained from the fit.

**Figure 5:**
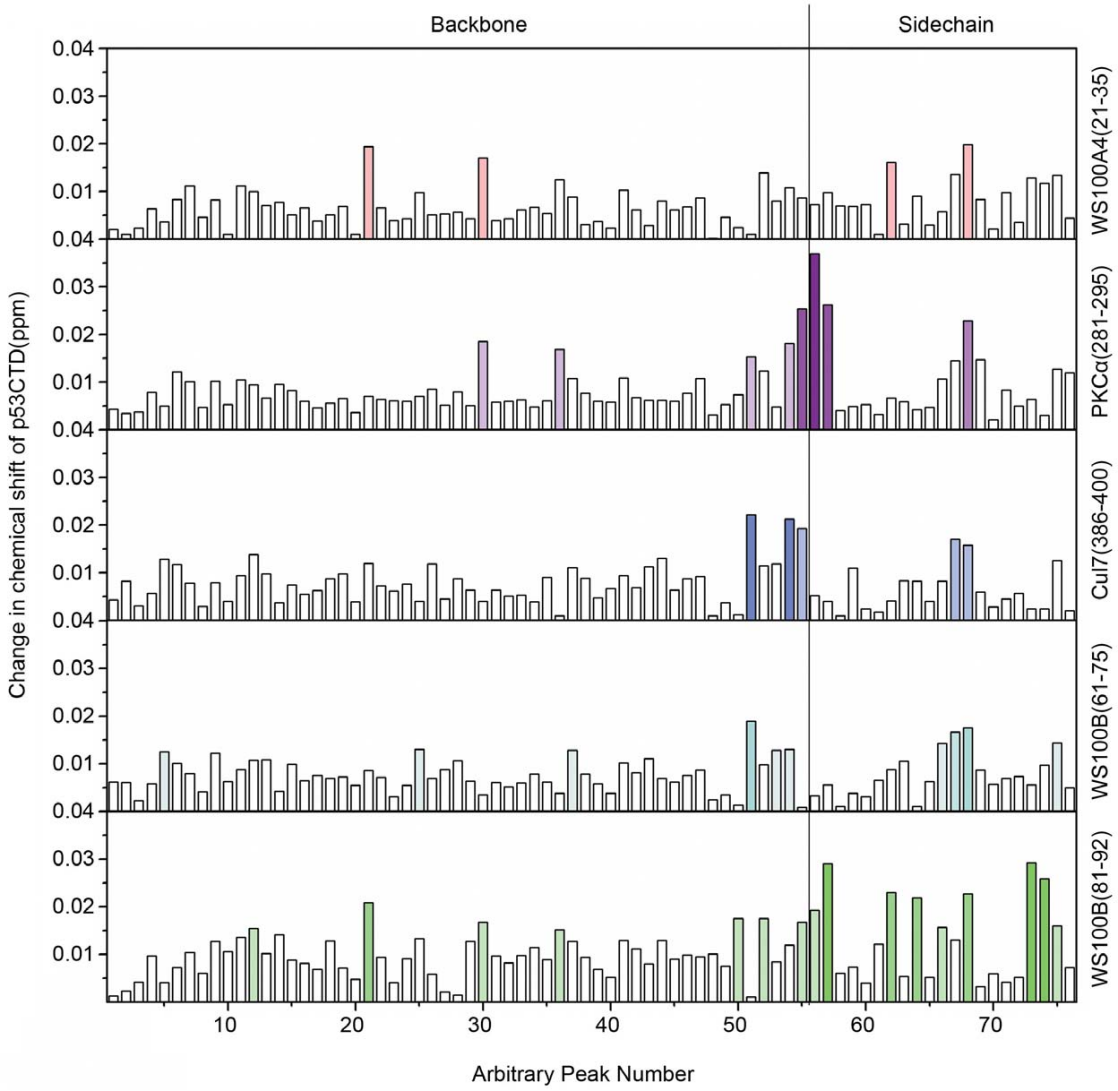
Changes in chemical shifts of ^15^N-labeled p53CTD upon incubation with various peptides. ^ 15^N–^1^H HSQC NMR spectra of the protein were measured with and without peptide, and the changes in chemical shifts were calculated as (δΔ^1^H^2^+(δΔ^15^N/5)^2^)^0^.^5^. The left panel shows the chemical shift deviations for backbone amide nitrogen atoms, and the right panel shows the chemical shift deviations for side chain nitrogen atoms. Numbers on the x-axis are arbitrary serial numbers for peaks and are unrelated to residue sequence.

We performed ^15^N–^1^H HSQC-NMR experiments to characterize the binding of the peptides to ^15^N-labeled p53CTD. Chemical shift deviations upon binding to p53CTD were determined for four representative binding peptides and for the non-binding peptide WS100A4(21–35) as a control. The deviations are plotted against arbitrarily assigned peak numbers in figure 5. The overlay of the ^1^H–^15^N amide region and the side chain peaks is given in Figure S2. All four peptides showed small chemical shift deviations in a subset of residues. More significant deviations were observed for the peptides PKCα(281–295) and WS100B(81–92), especially in the side chain peaks. Side chain nitrogen chemical shifts are very sensitive to changes in buffer conditions, but comparison to the control peptide WS100A4(21–35), which had a small effect on the side chain peaks, indicates that the observed deviations are due to peptide binding. All binding peptides induced chemical shift deviations at peaks 50–58 and 66–68, in addition to some specific deviations, indicating a partial overlap between the binding sites of the different peptides.

### PKCα(281–295) Mildly Stabilizes p53CTD

**Figure 6:**
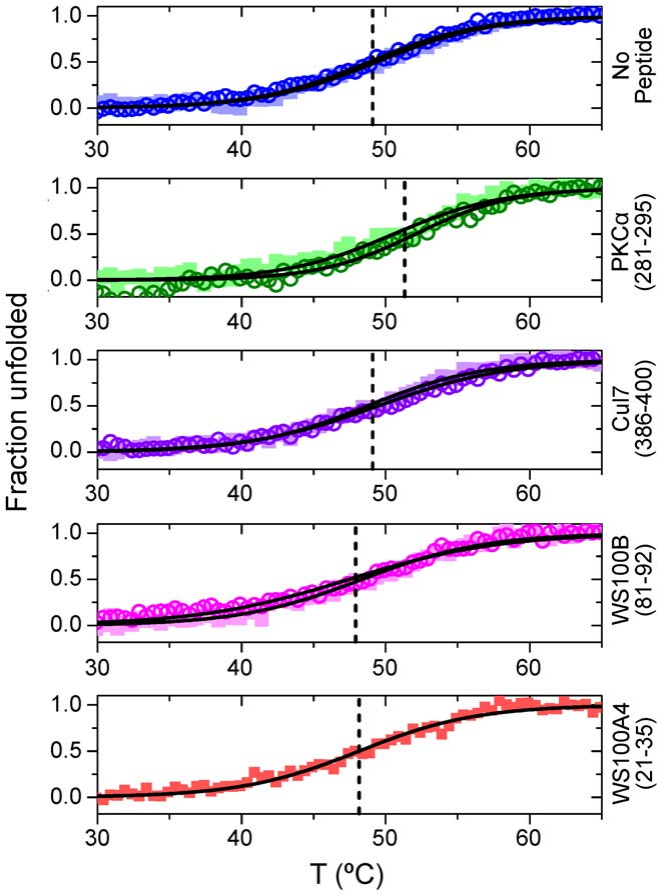
Thermal denaturation curves of p53CTD L344A in the presence of peptides from the array. 20 µM protein was heated from 25°C to 65°C with or without 100 µM peptide. The data were fit to a sigmoidal curve describing a transition between two states. Raw data for two repeats (squares and hollow circles) are shown in the background. The dashed lines represent the averaged melting temperatures for both repeats.

To test the effects of the binding peptides on the stability of the p53CTD tetramer, we used circular dichroism and followed the effect of the peptides on the structural changes upon thermal denaturation of p53CTD. It was previously shown that upon heating the p53 tetramerization domain, it undergoes dissociation of the tetramer coupled to denaturation of the monomers [41]. However, since p53CTD is already fully tetrameric at concentrations above 1 µM, we used the mutant protein p53CTD L344A, which has a dimer-tetramer dissociation constant of ∼20 µM (data not shown). The peptide PKCα(281–295) showed a small but significant increase in the melting temperature of *ΔT_m_* = 2.2±1.0°C (Figure 6, Table 4).

**Table 4:** Melting temperatures of p53CTD L344A in the presence of peptides from the array.

Peptide	T_m_ (°C)
No peptide	49.1±0.4*^A^*
Cul7(386–400)	49.1±0.5
PKCα(281–295)	51.3±1.0
WS100B(81–92)	47.9±0.5
WS100A4(21–35)	48.2±0.1

*A:* The errors indicated are the deviation from the average of 2 repeats, except for WS100A4, where the error indicated is the standard error obtained from the sigmoidal fit.

### The Peptides Bind Specifically to p53 Tetramers

We tested whether the peptides that bind tetrameric p53CTD can bind specifically to tetrameric p53 in a mixture of the different oligomers. Testing the specificity of the peptides for tetrameric p53 must be performed at very low p53 concentrations (<100 nM), where a significant population of dimers is present in addition to the tetramers [21]. Therefore, we used fluorescence-monitored analytical ultracentrifugation sedimentation velocity experiments using FlAsH-labeled full-length p53. The fluorescent label on p53 enables working at nanomolar protein concentrations and allows for specific detection of the protein without interference from any other molecule in the solution. This method has been extensively used to characterize the oligomerization equilibrium of p53 and related proteins alone and with binding proteins [21], [24], [29].

**Figure 7:**
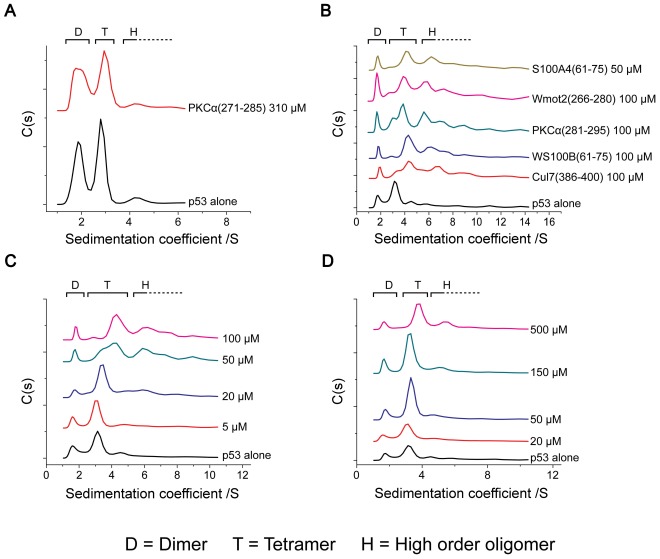
Sedimentation profiles of FlAsH-labeled p53 (25–50 nM) measured in the presence of the peptides. A) PKCα(271–285) had no significant effect on the profile. B) Peptides that shifted the tetramer peak. C) Concentration dependent behavior of the peptides. Sedimentation profiles were measured with different concentrations of WS100B(61–75). Similar behavior was observed for the other peptides in B (data not shown). D) Behavior of Cul7(376–390). This peptide binds p53 only weakly, and causes a shift of the tetramer peak at very high peptide concentrations. Similar behavior was observed for PARC(386–400).

We incubated FlAsH-p53 with different concentrations of the peptides and measured the sedimentation profiles of the protein (Figure 7). To identify the oligomeric state which preferentially interacts with the peptides, we used the fact that p53 tetramers exchange very slowly [42], with a *t*_1/2_ of several hours at 20°C, and even slower at 10°C used in our experiments. Effectively, there was little re-equilibration during the experiment, and only oligomeric states interacting with the peptides were affected. Sedimentation coefficients of∼2 S correspond to dimers and∼3 S correspond to tetramers. Presumably, sedimentation coefficients of up to∼4 S correspond to p53 tetramers bound to one or more peptide molecules. While binding of a small peptide is unlikely to induce significant change in the molecular weight, it may induce a change in the shape of a molecule, leading to a more compact state with a higher sedimentation coefficient. Most likely higher sedimentation coefficients (>4 S) correspond to higher-order oligomers of p53. Table 2 summarizes the results of the AUC tests. The effect of the peptides is clearly related to their charge and affinity to the protein. Highly charged and tight binding peptides bound p53 tetramers at lower peptide concentrations and also caused aggregation at excess concentrations. Peptides with a low charge and low affinity had a small or no effect. Eight peptides caused the tetramer peak to shift or gradually disappear while higher p53 oligomers formed (Figure 7B). The dimer peak was unaffected. This indicates that the peptides bound mainly to the tetrameric fraction of the protein and some of them might have also induced the formation of small aggregates, or bridged two or more p53 tetramers. The behavior of the peptides was concentration-dependent (Figure 7C): At low peptide concentrations (20–50 µM) the tetramer peak started to shift, indicating binding of peptides to the protein. At higher peptide concentrations, the protein became saturated with bound peptides and high-order oligomers formed, the extent of aggregation increasing with peptide concentration. The only peptide that showed no effect was PKCα(271–285) (Figure 7A), presumably because its binding to p53CTD was weak (*K*_d_ = 105 µM) and we could not reach a concentration above 310 µM with it. For peptides that bound p53CTD weakly, such as PARC(386–400) and Cul7(376–390), a specific shift of the tetramer peak was observed but at much higher concentrations, and no high-order oligomers were formed (Figure 7D).

## Discussion

In this study we designed a peptide array derived from proteins known to interact with the C-terminal domain of p53 and screened them for binding recombinant p53CTD. We characterized the binding of the peptides to p53CTD and the dependence of the binding on the oligomerization state of full-length p53 by quantitative biophysical techniques, using purified proteins and peptides. The peptides we identified bound tetrameric p53CTD at distinct sites with affinities as low as 5 µM, and bound specifically to tetrameric full-length p53 in fluorescence-monitored analytical ultracentrifugation. The peptides bound p53CTD at defined sites as shown by NMR, and the peptide PKCα(281–295) increased the thermodynamic stability of the p53CTD tetramer, as shown by circular dichroism.

### Implications on the Protein-Protein Interactions of Tetrameric p53

The p53CTD-binding peptides identified in the array screening were all derived from proteins known to bind the p53CTD. The binding sites we identified for Cul7 and PARC are in agreement with existing data [30]. We also discovered the precise binding sites of p53CTD within PKCα and Mot2, which have not been reported previously.

**Figure 8:**
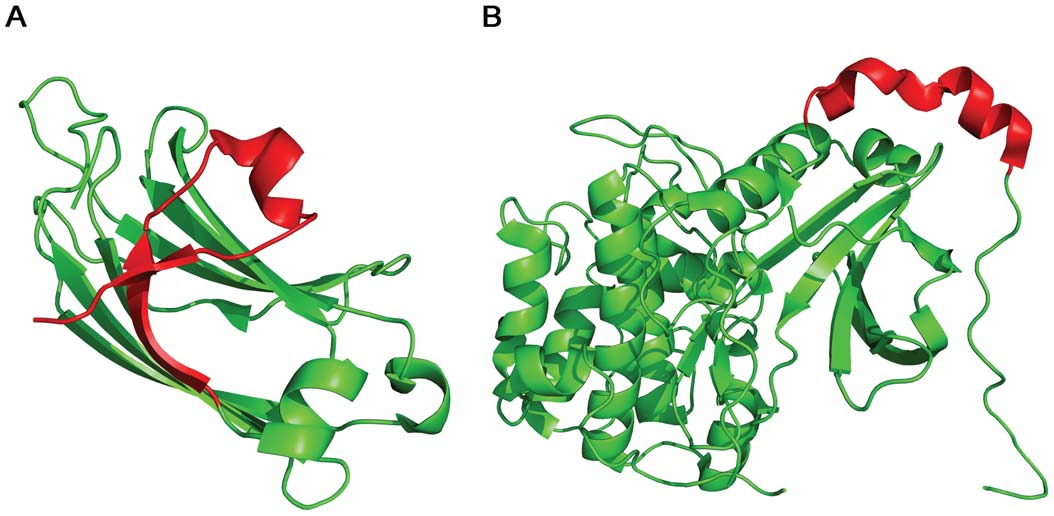
Binding sites for p53CTD on two PKCα domains. A) The C2 domain of PKCα (pdb 1dsy). Residues 271–292 are colored in red. B) The catalytic domain of PKCα (pdb 3iw4). Residues 641–655 are colored in red.

Our results demonstrate two separate p53CTD-binding sites in the protein kinase PKCα. The major binding site is located between residues 271–295 in the C2 domain of the protein. This is a calcium-dependent membrane-binding domain involved in the regulation of PKCα activity and is a hub for many protein-protein interactions of PKCα [43]. This site is well conserved among other members of the cPKC family (PKCβI, PKCβII and PKCγ) but not in other PKC proteins. It is located on a completely exposed, partially structured loop in the C2 domain [44] (Figure 8A). A weaker binding site is located between residues 641–655, on an exposed helix in the catalytic domain of PKCα [28], [43] (Figure 8B). This site is highly conserved among all PKC proteins. It is therefore possible that although all PKC proteins may bind p53CTD, only members of the cPKC family can bind p53CTD specifically and with high affinity.

**Figure 9:**
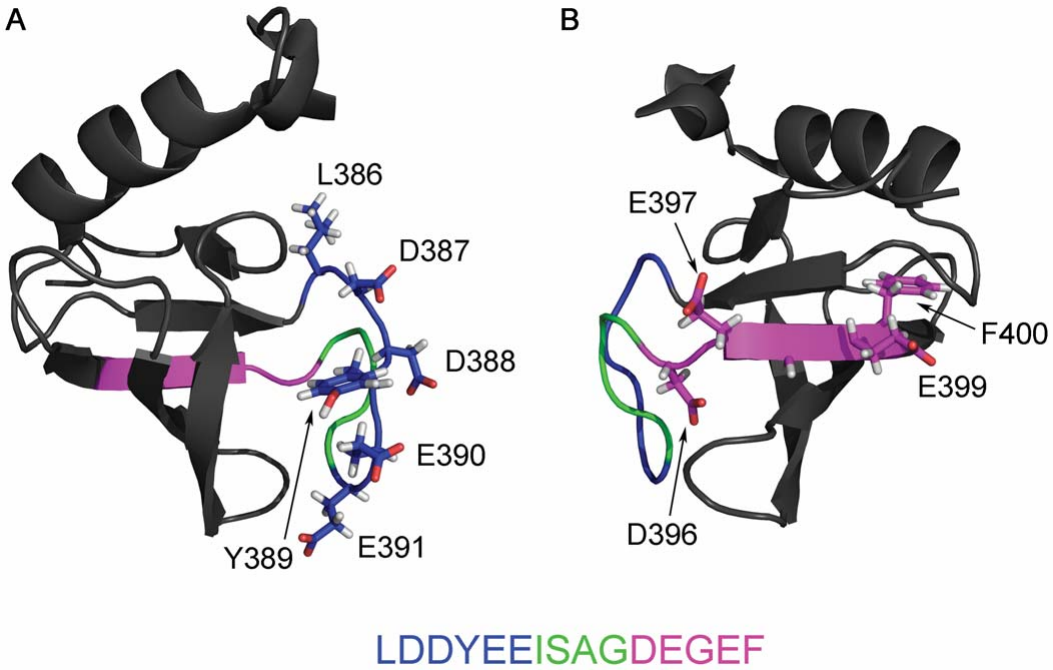
NMR structure (pdb 2jng) of the Cul7 CPH domain, residues 360–440. The binding site for p53CTD is color marked as indicated in the figure. The two views are rotated∼180° with respect to each other. A) Direct view of the LDDYEE motif; B) Direct view of the DEGEF motif.

Examination of the binding sites of Cul7, PARC and S100B to p53CTD provides insights about the various contributions to the interactions, as well as the possible dependence on the oligomeric state of p53. The E3 ubiquitin ligases Cullin7 and PARC are known to interact with p53 via the conserved CPH domain in their N terminal regions [31], and have been shown to restrict p53 to the cytoplasm and antagonize its function [30], [45]. Our results locate the interaction between p53CTD and a conserved region spanning residues 376–400 in Cul7 and a 100% homologous region in PARC, in excellent agreement with previously published data [30]. This region contains two motifs that are both highly charged and contain exposed hydrophobic and aromatic residues (Figure 9). As seen in the NMR structure of the CPH domain of Cul7 [31], the two motifs are fully exposed, face opposite directions and are perpendicular. We hypothesize that the two motifs may bind synergistically to different sites in p53CTD, or to different monomeric subunits in tetrameric p53CTD, resulting in a much higher affinity than each motif alone.

S100 proteins were shown to bind both the tetramerization domain and the negative regulatory domain of p53 with affinities in the micromolar range [22], [23]. All S100 proteins bind preferably to lower oligomeric forms of p53Tet. However, in our experiments p53CTD was predominately tetrameric, therefore preventing the detection of p53Tet-binding peptides derived from S100A4, and explaining the weak binding of the p53Tet-binding peptide derived from S100B (residues 81–92). Binding to the NRD is a less common feature of S100 proteins. S100B binds the NRD with a *K*_d_ of about 100 µM [23], while the binding of S100A4 to the NRD is much weaker, in agreement with our results. S100 proteins also bind the N-terminal transactivation domain of p53, and their affinity to p53CTD and the p53 N-terminal domain is strongly affected by post-translational modifications [24]. We speculate that the mechanism of binding between the proteins, and therefore the binding sites, may vary depending on the oligomerization state of p53 and other factors such as post-translational modifications [24].

The NMR results indicated that all four representative peptides bound p53CTD at defined sites that partially overlap, whereas the control peptide WS100A4(21–35) showed smaller, less localized chemical shift deviations. The peptides PKCα(281–295) and WS100B(81–92) showed more significant chemical shift deviations than the other peptides. This is consistent with our other results since PKCα(281–295) increased the thermodynamic stability of p53CTD, and WS100B(81–92) bound directly to the p53 tetramerization domain in the peptide array.

### The Peptides Bind Specifically to Tetrameric p53: Implications

The binding tests indicated that the peptides bound p53CTD in its tetrameric form. Notably, in the peptide array screening, the peptide PKCα(281–295) bound more tightly to the tetrameric full length p53CTD than to the disordered, monomeric negative regulatory domain (p53NRD). The AUC results confirmed these observations: for most of the peptides, the tetramer peak was shifted and even replaced by peaks of higher oligomers at excess peptide concentration, while the dimer peak remained unchanged, indicating that the peptides bind preferentially to the tetrameric fraction of the protein. The peptides that showed a weak or no effect were all weak binders that probably did not bind p53 tightly enough to exert an effect. The formation of high-order oligomers at high peptide concentrations was probably the result of non-specific aggregation mediated by the highly charged peptide molecules. Most peptides showed preferential binding to the p53 tetramer even though nine of the peptides bound the disordered p53NRD. This may indicate that the peptides can bridge several NRDs in the same tetramer, thus stabilizing the tetrameric state and by doing so causing tighter binding to tetrameric p53.

Many proteins reported to bind p53CTD gave no interaction in the array. Most of these proteins were previously shown to bind directly to the p53 tetramerization domain. These proteins include the HIV Tat protein, the protein BAF60a [46] which is part of the chromatin remodeling complex, the protein kinases cdc2 [28] and CK2β [27], and the transcription factor C/EBPβ [47]. The proteins S100B, S100A4, Mot2 and PKCα interacted very weakly or not at all with p53Tet in our experiments, despite previous reports of such interactions [23], [26], [38]. In general, we hardly observed any interactions between peptides in the array and p53Tet. This is probably because: (1) p53CTD was tetrameric at all concentrations used in this study. The hydrophobic dimer-dimer interface of p53Tet [14], [15] may interact with other proteins, as with the Exportin receptor [20]. However, this interface is only exposed in p53 dimers and monomers, and therefore proteins that bind it would not be detected in our screening; (2) The p53CTD construct we used contained no post-translational modifications, which are known to significantly affect the protein-protein interactions of p53 as well as its protein-DNA interactions [24], [29], [48], [49]. Therefore, in this study we identified a selection of peptides which preferentially bind the tetrameric state of p53 and do not require post-translational modifications for their activity.

The peptides prove the concept that preferential binding to p53 tetramers can be achieved. However, the effect of the peptides on p53 oligomerization and stability was small, as shown by the circular dichroism measurements. The AUC results indicate that many p53CTD-binding proteins bind specifically to tetrameric p53. Although the peptides bound preferably to p53 tetramers, the affinities of the peptides to p53 (>15 µM at physiological ionic strength) were relatively weak compared to the p53 dimer-tetramer dissociation constant and the affinity of p53 to DNA (which are both around 20 nM). However, the binding of the full-length proteins to p53 may be tight enough to significantly stabilize the active tetramer.

Tetramerization of p53 plays a crucial role in the anti-cancer activity of p53. Data from multiple sources [20], [23], [26], [27], [28], [38], [46] indicate that p53 may exist as dimer or monomer for significant times in the cell – otherwise the tetramerization domain would not be involved in so many interactions, and p53 would never be exported from the nucleus. Therefore, the transition from a predominately monomer/dimer population to an active tetrameric population is a critical stage in p53 activation. Our results indicate that the protein-protein interactions of p53 may be strongly involved in this transition: The transition may be stimulated by specific binding of proteins to tetrameric p53, or alternatively, the transition to a mostly tetrameric p53 population may induce the dissociation of previous interaction partners and the formation of new interactions, which may be important for the proper function of p53.

The specific binding of the peptides to tetrameric p53 may have future therapeutic potential. One of the peptides, PKCα(281–295), showed a slight stabilization of tetrameric p53 in circular dichroism measurements, and bound tighter to full-length tetrameric p53CTD than to the monomeric p53NRD in the peptide array screening. Although currently the effect of the peptides on p53 tetramer stability is, due to their low affinity, currently small, less charged compounds with higher affinities derived from these peptides may have a stronger effect on p53 oligomerization and activity.

## Materials and Methods

### Expression and Purification of p53CTD

The plasmid pRHislipoTEVp53CTD was constructed and the protein was expressed and purified as described [23]. For the expression of ^15^N-labeled p53CTD, C41 *E.Coli* cells were grown in MOPS minimal media [50] with ^15^N-enriched ammonium chloride, and grown at 37°C until the OD_600_ reached ∼0.8. The culture was then transferred to 17°C and 0.1 mM of isopropyl β-D-thiogalactoside was added to induce the expression of the protein. The cells were harvested after overnight incubation. The protein was purified using the same protocol as the non-labeled protein [23]. The L344A mutation was introduced into the plasmid using the QuikChange site directed mutagenesis kit (Strategene), and the mutant protein was expressed and purified using the same protocol as the wild type.

### Peptide Synthesis and Purification

Peptides were synthesized on a Liberty peptide synthesizer with a Discover single mode microwave module, using standard Fmoc chemistry. Protected amino acids were purchased from Luxembourg Bio Technologies (Tel Aviv, Israel), Iris Biotech GmbH (Marktredwitz, Germany), and Chem-Impex (Wood Dale, IL, USA). For fluorescein labeling, the peptidyl resin was reacted with 5(6)-carboxyfluorescein (Molecular Probes™) as described [51]. Peptides were cleaved from the resin following a standard procedure [33]. The peptides were purified on a Vydac C8 semipreparative column using gradients of 5% to 60% acetonitrile in water, with 0.1% trifluoroacetic acid (TFA) in both solvents. The mass of the peptides was measured using an Applied Biosystems Voyager-DE Pro MALDI TOF mass spectrometer and verified to be within ±1 Da of the theoretical mass. The purity of all peptides was verified to be >95% for non-labeled peptides and >90% for fluorescein-labeled peptides by analytical HPLC. The purified peptides were lyophilized from 30% acetic acid to remove residual TFA. The concentrations of the peptides were measured by UV absorbance at 280 nm using extinction coefficients of 1490 M^−1^cm^−1^ for tyrosine and 5500 M^−1^cm^−1^ for tryptophan. For peptides with no tyrosine or tryptophan in the sequence, a single tryptophan residue was added at the N-terminus. For fluorescein-labeled peptides, absorbance was measured at 495 nm with an extinction coefficient of 65000 M^−1^cm^−1^.

### Peptide Array Screening

Celluspots™ peptide arrays were purchased from Intavis (Köln, Germany). The array was blocked for one hour at room temperature with 2% skimmed milk in TBS-T (50 mM Tris pH = 7.5, 110 mM NaCl, 0.05% Tween 20), and washed three times with TBS-T. p53CTD was diluted in 1% milk in TBS-T to 20 µM. The protein was incubated with the array overnight at 4°C. The array was washed three times with TBS-T. Protein was detected using the FL-393 anti-p53 antibody (Santa Cruz). For identifying the binding site of each peptide within p53CTD, the blocking step was performed as described above, and His_6_–p53(326–355) (His_6_-p53Tet) or His_6_-W-p53(361–393) (His_6_–p53NRD) were diluted to 20 µM in 1% milk in TBS-T, and incubated with the array overnight at 4°C. The array was then washed three times with TBS-T and proteins detected using the His-probe HRP-conjugated monoclonal antibody (Santa Cruz).

### Fluorescence Anisotropy Binding Studies

Binding tests were performed in 20 mM sodium phosphate buffer, pH = 7.2, 13.5 mM NaCl. Measurements were performed at 10°C using a PerkinElmer LS-55 luminescence spectrofluorimeter equipped with a Hamilton microlab 500 dispenser [52]. The fluorescein-labeled peptides were dissolved in buffer and diluted to a final concentration of 100 nM. 800 µl of the labeled peptide solution were placed in a cuvette, and the protein (200 µl∼1 mM monomer, dialyzed into the buffer) was titrated into the labeled peptide in aliquots of 4–10 µl with 30 seconds mixing and 1 minute intervals. The total fluorescence and anisotropy were measured after each addition using an excitation wavelength of 480 nm and an emission wavelength of 530 nm. The data were fit to the following single-site model equation (equation 2):
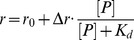
(2)where 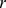 is the measured fluorescence anisotropy value, 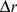 is the amplitude of the fluorescence anisotropy change from the initial value (peptide only) to the final value (peptide in complex), 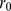 is the starting anisotropy value corresponding to the free peptide, 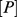 is the protein concentration, and 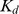 is the dissociation constant. For peptides that contain a cysteine in the sequence, 5 mM β-mercaptoethanol was added to the buffer to ensure the peptide is in its monomeric form during the titration.

Ionic strength dependence studies were conducted as above with the following minor changes: 200 µl of protein stock were titrated to 800 µl of 200 nM peptide. The buffer was 10 mM sodium phosphate, pH = 7.2 (ionic strength = 18 mM). The ionic strength of the buffer was modified by adding varying amounts of 2M NaCl in 10 mM sodium phosphate, pH = 7.2. The temperature was 25°C. Dissociation constants obtained were plotted against saline solution activity, taken from previously published data [53], and fit linearly.

### NMR Studies

NMR studies were conducted in 20 mM NaP_i_, pH = 7.2, 13.5 mM NaCl, 2 mM beta-mercaptoethanol, and 10% D_2_O. ^15^N–p53CTD and the peptides were dialyzed using GeBAflex tubes with a MWCO of 1 kDa (Gene Bio-Application ltd., Israel) against the experimental buffer. Samples contained 200 µM ^15^N–p53CTD and 400 µM peptide. For the less soluble WS100B(81–92), the sample contained 108 µM ^15^N–p53CTD and 216 µM peptide. NMR spectra were recorded using a Bruker Avance 600 MHz DMX spectrometer operating at the proton frequency of 600.13 MHz using a 5 mm selective probe equipped with a self-shielded xyz-gradient coil. Experiments were conducted at 278 K using standard Bruker pulse sequences. Spectra were processed and analyzed with the TopSpin software package (Bruker Analytische GmbH) and SPARKY (T.D. Goddard and D.G. Kneller, SPARKY 3, University of California, San Francisco). Changes in chemical shift were calculated as (δΔ^1^H^2^+(δΔ^15^N/5)^2^)^0.5^ where Δ^1^H and Δ^15^N represent the peak offsets in ppm in the presence of the peptides, for the hydrogen and nitrogen dimensions, respectively [54].

### Circular Dichroism Thermal Denaturation Measurements

CD spectra were recorded on a JASCO J-810 Spectrophotometer (JASCO, Japan) equipped with a Peltier thermostat using the supplied SpectraManager software. The samples contained 20 µM p53CTD L344A in 25 mM phosphate buffer, pH = 7.2 with or without the addition of 100 µM peptide. The sample was heated at a rate of 50°C per hour, from 25°C to 65°C and the CD reading was taken at λ = 222 nm every 0.1°C with a response time of 8 s per point. Full CD spectra of the sample (190–260 nm) were measured at 25°C before and after the experiment to confirm the reversibility of the denaturation process. Background scans were conducted on the peptides in buffer alone, and subtracted from the protein + peptide scans. The fraction of unfolded tetramer *F_u_* was calculated from the ellipticity by the relationship:
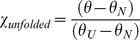
(3)where *θ* is the value of ellipticity at any temperature, and *θ_N_* and *θ_U_* represent the ellipticity values at the temperature where the fully folded and fully unfolded states exist, respectively. The data were then fit using a sigmoidal model, and *T*_m_ was defined as the temperature at half height.
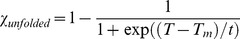
(4)where t is a constant describing the slope of the melting curve.

### Fluorescence Monitored Analytical Ultracentrifugation

The studies were performed with a quadruple mutant of p53, (M133L/V203A/N239Y/N268D) [55], which is significantly more stable than wild-type p53 but has similar activity and DNA-binding properties. C-terminally FlAsH-tagged (CCPGCC) p53 was expressed and reacted with FlAsH-EDT_2_ as described [24]. This introduces a specific fluorescent label at the carboxy terminus of p53. The protein was dialyzed against 25 mM NaP_i_ pH = 7.2, 150 mM NaCl, 14.2 mM β-mercaptoethanol, 0.2 mg/ml BSA and 10% glycerol. The experiments were run with a protein concentration of 25–50 nM and with peptide concentrations of up to 500 µM or to the solubility limit of the peptide. The measurements were performed in a Beckman-Coulter Optima XL-I ultracentrifuge with an Aviv fluorescence detection system [29] in SedVel60K-FDS fluorescence velocity cells. The samples were equilibrated in the cells at 10°C for 3 hours before centrifugation. The AUC run was performed as described [23], [48] and analyzed using SedFit software [56].

## Supporting Information

Figure S1 (TIF)

Figure S2 (TIF)

Table S1 (PDF)
