# Supplementary figures and images for: Specific Recognition of p53 Tetramers by Peptides Derived from p53 Interacting Proteins
Source: PLoS One. 2012 May 31;7(5):e38060. doi: 10.1371/journal.pone.0038060 (PMC3365014; doi:10.1371/journal.pone.0038060)

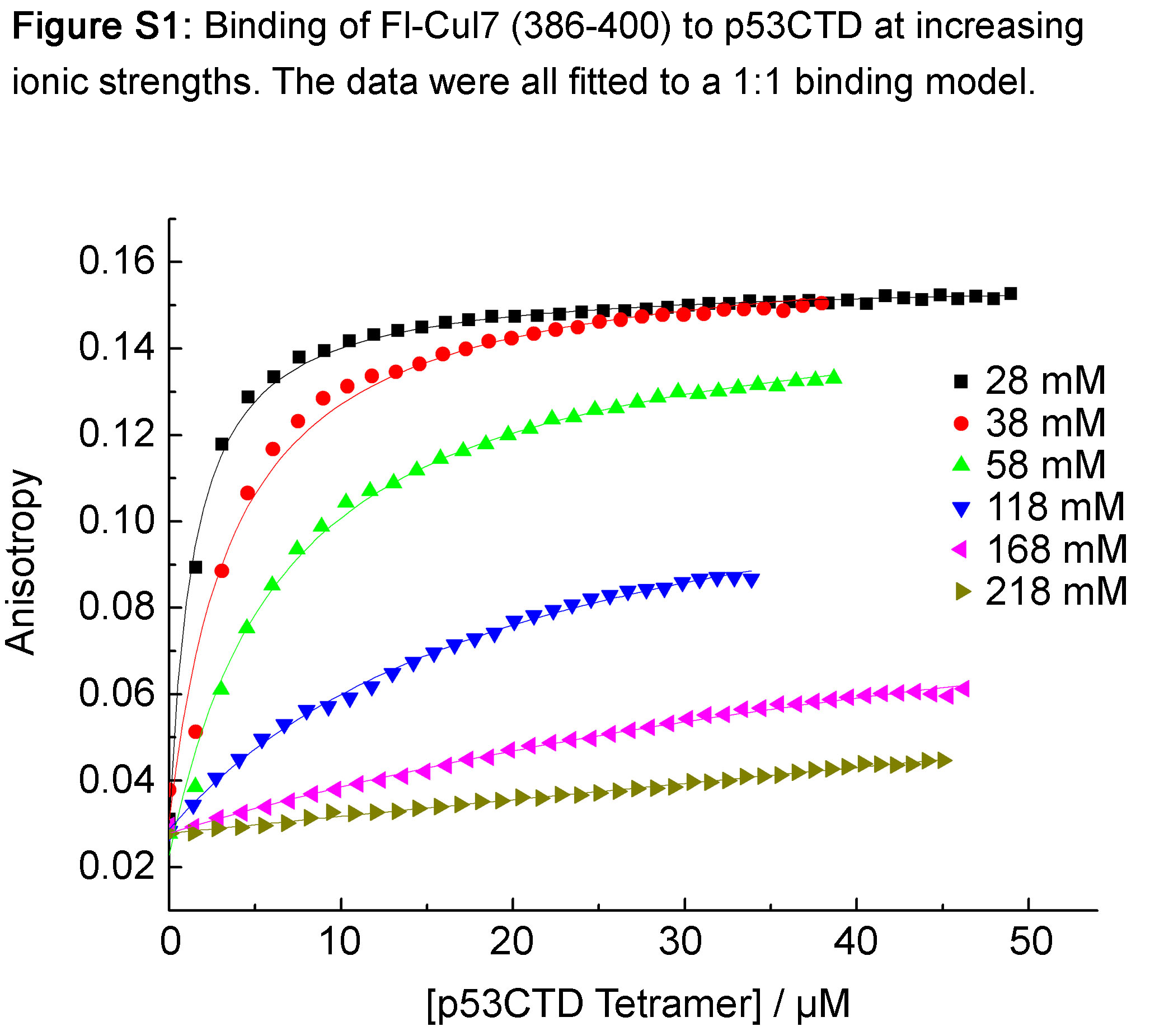

Supplement: Figure S1 — (TIF) [file pone.0038060.s001.tif]

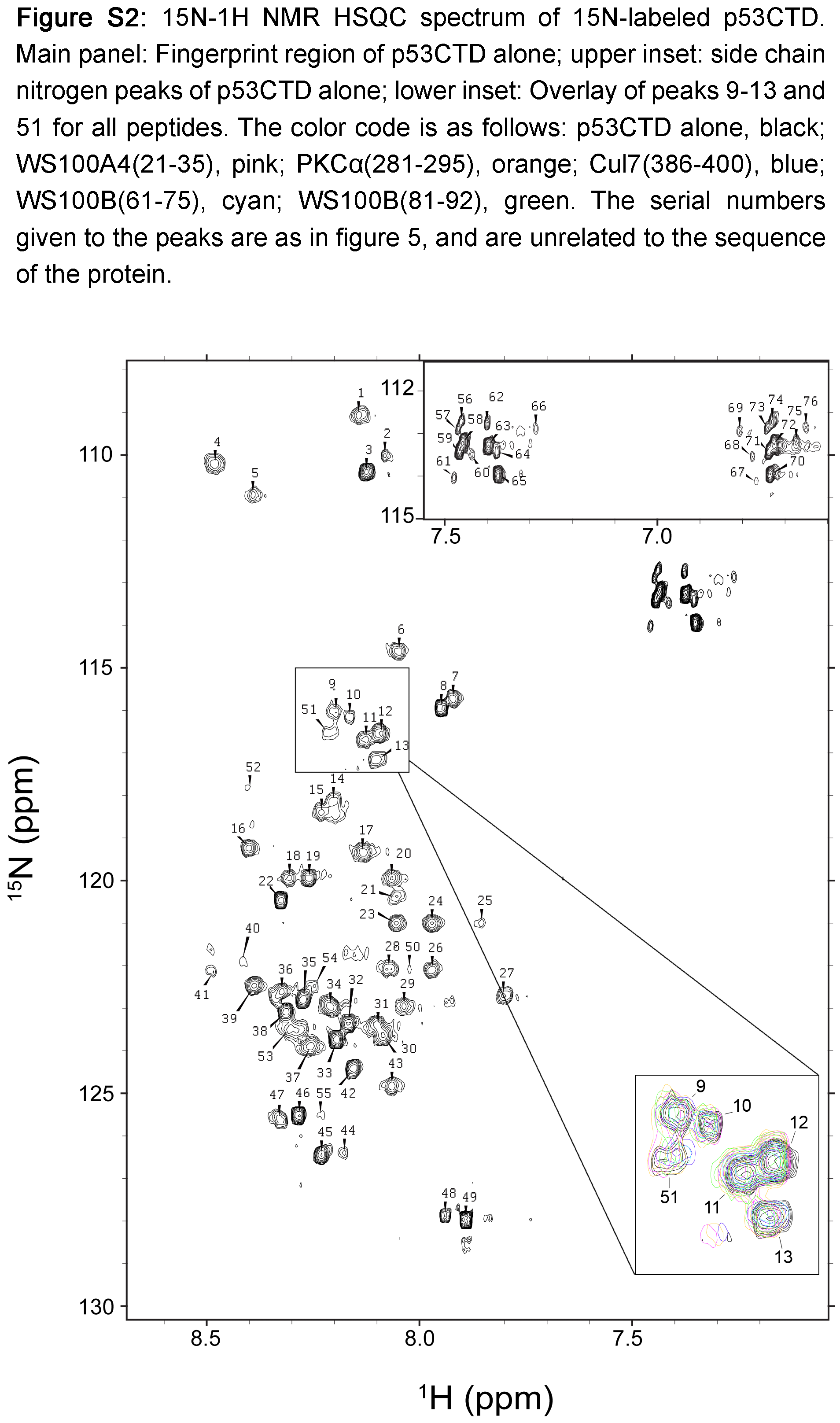

Supplement: Figure S2 — (TIF) [file pone.0038060.s002.tif]
