# Supplementary material for: Specific Recognition of p53 Tetramers by Peptides Derived from p53 Interacting Proteins
Source: PLoS One. 2012 May 31;7(5):e38060. doi: 10.1371/journal.pone.0038060 (PMC3365014; doi:10.1371/journal.pone.0038060)
Supplement: Table S1 — (PDF) [file pone.0038060.s003.pdf]

Table S1: Proteins tested in the peptide array			
Protein	Function/family	p53-Interacting domain	Reported interaction with p53
Cul7 [1,2]	E3 ligase	CPH domain (residues 360-460)	Represses p53-mediated transcription
PARC [2,3]	E3 ligase	CPH domain (366-466)	Inhibits p53 by sequestering it to the cytoplasm
P34^{cdc2} [4]	Protein kinase	Catalytic subunit	Phosphorylates Ser315 in p53. Binds residues 330-339 in p53.
Casein Kinase 2 [5]	Protein kinase	Residues 72-149 in the catalytic subunit	Phosphorylates p53 at Ser392. Binds residues 325-344 in p53.
PKCα [6]	Protein kinase	Not characterized	Phosphorylates p53 in the region 357-381. Binds residues 320-346 in p53.
S100B [7,8,9,10,11]	S100 family	Residues 40-60 and 75-90 in S100B by NMR	Binds the p53 C terminus. Inhibits PKC-mediated phosphorylation
S100A4 [9,11]	S100 family	Not characterized	Binds weakly to the p53 CTD and to tetrameric full length p53
Mot2 [12,13]	Heat shock protein	Residues 253-282 in Mot2	Binds to residues 312-352 in p53
BAF60a [14]	Part of the SWI/SNF chromatin remodeling complex	Residues 108-150 in BAF60a	Binds the p53 tetramerization domain. p53 recruits the SWI/SNF complex via binding to BAF60a.
C/EBPβ [15]	Transcription factor	Residues 199-345 in C/EBPβ	Binds residues 326-393 in p53 and inhibits p53.
HIV-1 Tat [16,17]	Transcription of viral genes	Residues 1-34 and 47-57	Binds the p53 tetramerization domain

1. Andrews P, He YJ, Xiong Y (2006) Cytoplasmic localized ubiquitin ligase cullin 7 binds to p53 and promotes cell growth by antagonizing p53 function. Oncogene 25: 4534-4548.
2. Kaustov L, Lukin J, Lemak A, Duan S, Ho M, et al. (2007) The conserved CPH domains of Cul7 and PARC are protein-protein interaction modules that bind the tetramerization domain of p53. J Biol Chem 282: 11300-11307.
3. Nikolaev AY, Li M, Puskas N, Qin J, Gu W (2003) Parc: a cytoplasmic anchor for p53. Cell 112: 29-40.
4. Wagner P, Fuchs A, Gotz C, Nastainczyk W, Montenarh M (1998) Fine mapping and regulation of the association of p53 with p34cdc2. Oncogene 16: 105-111.
5. Gotz C, Scholtes P, Prowald A, Schuster N, Nastainczyk W, et al. (1999) Protein kinase CK2 interacts with a multi-protein binding domain of p53. Mol Cell Biochem 191: 111-120.
6. Delphin C, Huang KP, Scotto C, Chapel A, Vincon M, et al. (1997) The in vitro phosphorylation of p53 by calcium-dependent protein kinase C--characterization of a protein-kinase-C-binding site on p53. Eur J Biochem 245: 684-692.
7. Baudier J, Delphin C, Grunwald D, Khochbin S, Lawrence JJ (1992) Characterization of the tumor suppressor protein p53 as a protein kinase C substrate and a S100b-binding protein. Proc Natl Acad Sci U S A 89: 11627-11631.
8. Fernandez-Fernandez MR, Rutherford TJ, Fersht AR (2008) Members of the S100 family bind p53 in two distinct ways. Protein Sci 17: 1663-1670.
9. Fernandez-Fernandez MR, Veprintsev DB, Fersht AR (2005) Proteins of the S100 family regulate the oligomerization of p53 tumor suppressor. Proc Natl Acad Sci U S A 102: 4735-4740.
10. Rustandi RR, Drohat AC, Baldisseri DM, Wilder PT, Weber DJ (1998) The Ca(2+)-dependent interaction of S100B(beta beta) with a peptide derived from p53. Biochemistry 37: 1951-1960.
11. van Dieck J, Fernandez-Fernandez MR, Veprintsev DB, Fersht AR (2009) Modulation of the oligomerization state of p53 by differential binding of proteins of the S100 family to p53 monomers and tetramers. J Biol Chem 284: 13804-13811.
12. Wadhwa R, Yaguchi T, Hasan MK, Mitsui Y, Reddel RR, et al. (2002) Hsp70 family member, mot-2/mthsp70/GRP75, binds to the cytoplasmic sequestration domain of the p53 protein. Exp Cell Res 274: 246-253.
13. Kaul SC, Reddel RR, Mitsui Y, Wadhwa R (2001) An N-terminal region of mot-2 binds to p53 in vitro. Neoplasia 3: 110-114.
14. Oh J, Sohn DH, Ko M, Chung H, Jeon SH, et al. (2008) BAF60a interacts with p53 to recruit the SWI/SNF complex. J Biol Chem 283: 11924-11934.
15. Schneider-Merck T, Pohnke Y, Kempf R, Christian M, Brosens JJ, et al. (2006) Physical interaction and mutual transrepression between CCAAT/enhancer-binding protein beta and the p53 tumor suppressor. J Biol Chem 281: 269-278.
16. Longo F, Marchetti MA, Castagnoli L, Battaglia PA, Gigliani F (1995) A novel approach to protein-protein interaction: complex formation between the p53 tumor suppressor and the HIV Tat proteins. Biochem Biophys Res Commun 206: 326-334.
17. Gabizon R, Mor M, Rosenberg MM, Britan L, Hayouka Z, et al. (2008) Using peptides to study the interaction between the p53 tetramerization domain and HIV-1 tat. Biopolymers.
